# Options for waterpipe product regulation: A systematic review on product characteristics that affect attractiveness, addictiveness and toxicity of waterpipe use

**DOI:** 10.18332/tid/125079

**Published:** 2020-08-25

**Authors:** Anne S. Kienhuis, Reinskje Talhout

**Affiliations:** 1 Centre for Health Protection, National Institute for Public Health and the Environment, Bilthoven, The Netherlands

**Keywords:** waterpipe, health effects, product regulation, smoking products, waterpipe device characteristics

## Abstract

**INTRODUCTION:**

Despite its known adverse effects on human health, waterpipe smoking is increasing in popularity worldwide. However, compared to cigarettes, regulation of waterpipe product smoking lags behind and presents unique challenges. In search for regulatory options, this systematic review analyzes 36 studies on the differential effects on human health of the waterpipe characteristics including smoking products, heating sources, device components, and packages.

**METHODS:**

A systematic review was performed according to PRISMA guidelines, revealing 443 unique citations. After screening, 36 studies were included in the results. Research articles were selected to inform on differential effects caused by product characteristics on adverse health effects, attractiveness, addictiveness and prevalence of waterpipe use.

**RESULTS:**

Flavors are the key aspect that defines attractiveness of waterpipe product smoking. All waterpipe products, with or without nicotine, produce toxicants in similar quantities as cigarettes. Heating sources for waterpipe tobacco include charcoals and electrical heating. Both heating sources increase toxicant emissions in different ways. Hoses and mouth tips are device components that are often shared in waterpipe smoking. Sharing influences attractiveness by enriching the social experience. At the same time, it influences the transfer of infectious diseases by bacteria and viruses from one smoker to another. Studies showed that more generic and less attractive packages with health warnings are effective in reducing the attractiveness of waterpipe smoking.

**CONCLUSIONS:**

Based on our findings, we advise to include all waterpipe products, tobacco and non-tobacco, with or without nicotine, in tobacco product regulations and smoking bans in order to: ban waterpipe molasses with characterizing flavors or ban the use of flavorings at any level; mandate dissemination of information on all waterpipe tobacco elements to the national regulator; prescribe testing to regulate contents of waterpipe smoking products and heating sources. Moreover, we advise to stimulate research on emissions of waterpipes.

## INTRODUCTION

The waterpipe, also known as ‘shisha’ or ‘hookah’, is an instrument for smoking waterpipe tobacco and flavored herbs. When tobacco is used in the waterpipe, smokers are exposed to nicotine. Molasses that only consist of flavored herbs are generally nicotine-free. In this paper, we will refer to ‘waterpipe product’ as a collective term that includes tobacco and flavored herbs, with or without nicotine. We will refer to the act of waterpipe smoking as ‘waterpipe product smoking’ (WPS). In line with this, ‘waterpipe product’ and ‘waterpipe product smoke’ will refer to both tobacco and flavored herbs, with or without nicotine. WPS is gaining in popularity worldwide. It is most prevalent in Eastern Mediterranean and European countries and more popular among youth than adults^1^. The waterpipe is typically used in special cafés and it is culturally associated with relaxation and pleasant social interaction^2^. WPS results in the exposure of smokers and bystanders to the same harmful smoke components found in cigarette smoke^3^. When molasses with nicotine are smoked, WPS may become addictive as well^4^. The increasing popularity of WPS is associated with the extensive variety of flavored waterpipe products, which is attractive to youth^5^. This popularity in combination with its toxic and addictive potential comes with serious health risks; it is associated with heart diseases, pulmonary infections and various types of cancer^6,7^. Despite these associations, waterpipe users typically see WPS as less harmful and less addictive than cigarettes^8,9^.

There is a worldwide combat against the use of all tobacco products, formalized in the World Health Organization (WHO) Framework Convention on Tobacco Control (FCTC). The FCTC provides a framework that applies to all forms of tobacco. However, many countries have not yet implemented measures specifically for waterpipe tobacco. For non-cigarette products, including those for the waterpipe, there is an increased awareness about the importance of tobacco control measures at the country, regional and global level, as they may eventually result in significant public health benefits^10^. Waterpipe products are also regulated by the European Tobacco Products Directive^11^ but not as strongly as cigarettes. For example, waterpipe product packages now should carry only a text warning instead of a combined graphic and text health warning, and the use of characterizing flavors is still allowed. Regulation of a non-cigarette product such as waterpipe molasses with tobacco and/or flavored herbs does create unique challenges^12^. The endless combinations of waterpipe product characteristics, such as variety in non-flavored molasses with or without nicotine, heating sources (e.g. charcoal, steam stones, electric heating), base liquids, including the wide variety of device components and packages, can all influence the composition of and exposure to waterpipe tobacco smoke emissions, and hence their effect on human health.

The aim of the current paper is to review waterpipe product characteristics with respect to WPS attractiveness, addictiveness, and toxicity for users and bystanders. Two questions were addressed: 1) ‘Do variations in waterpipe products influence attractiveness, addictiveness, and toxicity of users and bystanders?’, and 2) ‘How are the findings in this study useful for waterpipe product regulations?’. As such, we analyze literature on variations in waterpipe products, heating sources, device components, and packages, and their potential effects on human health. Based on our findings, we make evidence-based recommendations for regulation of these waterpipe product characteristics for the protection of public health.

## METHODS

The search strategy, developed for the purpose of this systematic review, aimed to retrieve articles focusing on effects of design characteristics of waterpipe products and their effect on human health (users and bystanders). Research articles were selected to inform on differential effects caused by product characteristics on adverse health effects, attractiveness, addictiveness, and prevalence of waterpipe use. Records were included that were published in the period from 1 January until 1 September 2019 in the bibliographic database Embase, using the following items: ‘waterpipe tobacco smoking’, ‘hookah’, ‘shisha’, ‘risk’, ‘toxicity’, ‘flavor’, ‘molasses’, ‘herbal’, ‘warning’, ‘packaging’, ‘regulation’, and ‘policy’ (see Supplementary file, Table S1 for the full search strategy).

**Figure 1:**
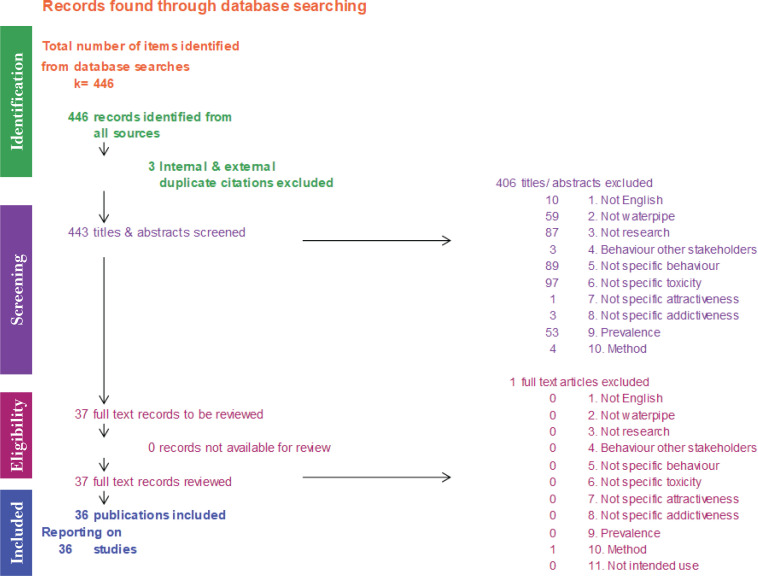
PRISMA flow diagram

Following the Preferred Reporting Items for Systematic Reviews and Meta-analysis (PRISMA) guidelines (Figure 1), 446 retrieved citations were screened, duplicates were eliminated, and the remaining 443 citations were organized in Endnote^13^. The two authors independently reviewed these titles using a previously agreed-upon exclusion criteria list. First, they independently screened a random sample of 66 titles and abstracts in which they were blinded to the article authors and journal titles, and reached strong agreement (Cohen’s Kappa=0.85)^14^. Second, the two authors independently screened all 443 titles and abstracts, still blind to authors and journal titles, using an Excel workbook designed specifically for this screening^15^. Exclusion criteria were in hierarchical order, meaning that if the first exclusion criterion applies, the other criteria were not checked. Exclusion criteria were the following: 1) The article was not in English; 2) The article was not about a waterpipe intended for smoking; 3) It was not a research paper; 4) The article was about behavioral research amongst other groups than waterpipe smokers or bystanders; 5) The article concerned behavioral research (e.g. attitudes towards waterpipe smoking) without any link to specific waterpipe design characteristics, heating source or package; 6) The article concerned health effects of the entire product category ‘waterpipe’ without links to specific waterpipe design characteristics, heating source or package; 7) The article concerned attractiveness of the entire product category waterpipe without link to any specific design characteristic, heating source, package; 8) The article concerned addictiveness (or cessation) of the entire product category waterpipe without link to any specific design characteristic, heating source, package; 9) The article concerned prevalence of the entire product category waterpipe without link to any specific design characteristic, heating source, package; and 10) The article only concerned method development.

The screening resulted in a total number of 37 full-text records^16^. To avoid outcome bias, full-text records were critically appraised by the independent reviewers for methodological quality and relevance using the Excel workbook^15^. This resulted in an additional exclusion of one publication. The remaining 36 studies were used for reporting in this systematic review.

## RESULTS

### Waterpipe products

The systematic search revealed 18 studies reporting on characteristics of waterpipe products that influence human health. Ten studies report on characteristics that increase the attractiveness of waterpipe smoking. Six studies report on toxicity of waterpipe product characteristics. One study reporting on attractiveness also specifically reports on the addictiveness of waterpipe smoking (Table 1).

**Table 1:** Waterpipe characteristics that influence human health

*Human health effect*	*Characteristics*
**Waterpipe products**	
Attractiveness	Flavors, nicotine content, price, socializing aspect, water in the bowl, taste, smell
Addictiveness	Nicotine
Toxicity	Waterpipe products and emissions, waterpipe tobacco and water waste
**Heating sources**	
Attractiveness	Charcoal nature (origin, quality)
Addictiveness	No characteristics found
Toxicity	Charcoal, electrical heating
**Device components**	
Attractiveness	No characteristics found
Addictiveness	No characteristics found
Toxicity	Mouthpiece
**Packaging, warning, and labelling**	
Attractiveness	Package, health warning
Addictiveness	No characteristics found
Toxicity	No characteristics found

#### Attractiveness

Four studies on the prevalence of WPS report flavor as the main reason to start and continue to use. Data from a longitudinal cohort study (2013–2014) in the United States on the Population Assessment of Tobacco And Health (PATH) showed that the majority of youth and young adult tobacco users consume tobacco products with characterizing flavors^17^. According to the definition in the European Tobacco Products Directive (EU-TPD), these are products with a clearly noticeable smell or taste, other than one of tobacco, such as fruit, alcohol or candy^11^. A cohort study showed that reasons for young adults to smoke waterpipe were the social aspect of waterpipe smoking and the fact that waterpipe tobacco comes in flavors they like^18^. A Canadian survey among students showed that just over half (51%) of the young adult waterpipe users reported use of flavored tobacco in the past 30 days^19^, while a survey in the United States among adult waterpipe users (aged ≥18 years) found this to be 87% across all sociodemographic groups, e.g. across age, race/ethnicity, education etc.^20^

Besides flavors, studies report other characteristics of WPS products that increase attractiveness such as the price of the tobacco product^21,22^ and nicotine content^22^. On average, participants liked waterpipe products with fruity flavors, low prices and labelled with low nicotine content, preferably nicotine-free^22^. Both ‘flavored’ and ‘nicotine-free’ labelled products were mostly preferred by females and non-smokers of cigarettes. The authors suggest that the liking of flavors and the fact that waterpipe tobacco manufacturers label their products as containing low levels of nicotine may have a gateway effect, especially on non-smokers of cigarettes^22^. Preferred waterpipe tobacco flavor increases the willingness to continue WPS^5^. Studies also suggest that flavored tobacco is a main reason for smoking initiation^23^, even in one case resulting in cigarette smoking^23,24^. Furthermore, characterizing flavors may prevent tobacco cessation among persons who may have otherwise quit^25^. Another main characteristic contributing to the attractiveness of waterpipe smoking is the socializing aspect^18,23,26^. This traditionally refers to smoking of the waterpipe with family or friends at home or in waterpipe smoking establishments. One study, however, reports a more recent phenomenon. In this study, Twitter posts on waterpipe were investigated, revealing online social communities around waterpipe, thereby notifying others about waterpipe-related social (real-life) events^26^.

Attractiveness of the flavored waterpipe products that produce smoke with a mild taste and pleasant smell in contrast to the harsher taste and smell of traditional tobacco, may increase preference for product use, thereby indirectly increasing toxicity. Especially, young adults prefer the mild taste and pleasant smell of smoke from flavored waterpipe tobacco and wrongly perceive the product as a less harmful or even harmless alternative to cigarette smoking^21,24^.

#### Addictiveness

Addictiveness of WPS is specifically addressed in one study and is attributed to nicotine containing waterpipe tobacco^5^. Authors expect that increased liking of tobacco flavor (i.e. attractiveness) stimulates use, thereby stimulating nicotine dependence.

#### Toxicity

The reviewed studies report on toxic constituents of various waterpipe products to which humans (users, bystanders) and/or the environment are exposed. These constituents were measured in waterpipe tobacco^27-30^, waterpipe tobacco smoke^30^, indoor air samples of waterpipe cafés^30,31^, and the waterpipe tobacco and water waste^32^. Constituents measured were: nicotine, tar, CO, trace metals, PAHs, aldehydes, and ultrafine particles.

One study analyzed nicotine and tar levels in three waterpipe products^27^. Tar and nicotine levels were compared to a large panel of tobacco products. Nicotine content in the waterpipe products was reported to be higher compared to levels in cigarettes, chewing tobacco and electronic cigarettes. Tar levels were reported to be lower in comparison with cigarette data, as reported by the United States Federal Trade Commission (FTC)^27^. The levels of nicotine and tar varied between the three waterpipe tobacco brands^27^.

Since metals are an important class of toxicants in tobacco products, the concentrations of eleven trace metals (Al, Mg, Ca, K, Cr, Mn, Fe, Cu, Ni, Zn, Sr) were analyzed in three popular waterpipe tobacco brands^28^. Results show that the analyzed waterpipe tobacco contains levels of metals that are in many cases higher than those in cigarettes^28^. The metal levels vary between the different brands^28^.

Besides waterpipe tobacco, which is the most common product smoked in the waterpipe, the smoking of flavored herbs in the waterpipe is increasing in popularity and has become widely available. These products are typically presented as ‘tobacco-free’ containing ‘0% nicotine’ and ‘0% tar’. Furthermore, these products are frequently referred to as ‘healthy’. This statement was invalidated by one study that analyzed the composition and the smoke emissions of three herbal waterpipe products and the air quality in establishments where they are smoked^30^. This study measured toxic trace metals, nicotine and polyaromatic hydrocarbons (PAHs) in unburned herbal tobacco. Furthermore, it compared toxicant emissions of CO, NO, tar, aldehydes and PAHs between three types of herbal tobacco and a popular brand of nicotine-containing waterpipe tobacco. Heavy metals and polyaromatic hydrocarbons (PAHs), known to be carcinogenic or possibly carcinogenic to humans, were found in unburned herbal products. Of even more concern were the quantities of toxicants including tar, PAHs, ultrafine particles and aldehydes that were found in the emissions of herbal products smoked under simulated conditions. These quantities equaled or exceeded concentrations found in emissions of nicotine containing waterpipe tobacco^30^. For example, tar levels were 909 mg/session for tobacco versus 2350 mg/session for one of the herbal samples.

Two studies on the indoor air quality of waterpipe cafés have analyzed the air quality in waterpipe cafés^30,31^ by analyzing BTEX (benzene, toluene, ethylbenzene, xylene)^31^ and particulate matter (PM), CO and nicotine^30^. Benzene concentrations were considerably higher than the occupational exposure limits, causing significant health effects such as cancer risk and chronic non-cancer hazards in particular for café workers who experience long-term exposure^31^. Both PM_2.5_ and CO levels were significantly higher in waterpipe cafés compared to cigarette smoking venues, whereas nicotine levels were mostly below detectable limits^30^. Selected cafés in this Canadian study were, according to tobacco smoking bans, officially only allowed to serve herbal products. According to the Canadian Air Quality Health Index (AQHI), the level of PM_2.5_ found in waterpipe cafés represents a potentially hazardous exposure. Overall, both studies show that waterpipe cafés have poor air quality and expose visitors to potential health hazards.

One study specifically addressed environmental pollution in relation to WPS and found small quantities of toxicants in the water waste from the bowl after waterpipe use. The study only investigated hazard and did not check whether the toxicant levels were sufficiently high to induce environmental health effects. However, the authors argue that environmental health risks may increase by the increasing popularity of waterpipe smoking^32^.

### Heating sources

The systematic review retrieved five research papers on the attractiveness^21^ and toxicity^33-36^ attributed to heating sources for WPS. The systematic search revealed no studies reporting on the addictiveness influenced by heating sources for WPS. The most commonly used heating source for waterpipe smoke is charcoal. A variety of charcoal can be used for waterpipe smoking. This includes charcoal from natural sources such as wood, or processed charcoal from wood or coconut origin to which chemicals and/or fuels are added to initiate the burning^33^. The popular quick-light charcoals are an example of such synthetic charcoals^36^.

#### Attractiveness

Charcoal quality was recognized as an important theme for visitors that reviewed waterpipe smoking establishments on the free online platform and social networking site Yelp (Yelp.com)^21^. Visitors preferred establishments were organic and natural charcoal were used. Low quality charcoal was one of the most negative factors in reviews of establishments; visitors referred to quick-light charcoals and reported to dislike them for their chemical flavour^21^.

#### Toxicity

Using charcoal as a heating source for WPS causes increased generation of CO in waterpipe tobacco smoke and is a main cause of CO poisoning in waterpipe smokers, of which severe cases have been observed in the last decade^36^. The type of charcoal used for heating matters; analysis showed that quick-light charcoal produces more than twice as much CO as natural charcoal^36^. Chemical analysis was performed on different brands of raw charcoal from natural and synthetic origin and on smoke generated from these brands^33^. Results showed the presence of toxic trace metals in raw charcoal samples and the generation of potentially harmful organic compounds in the smoke, identified by the authors as carcinogens, central nervous system depressants, and irritants^33^.

As such, electric heating has been proposed as a healthier alternative to charcoal heating. This was compared in two studies comparing chemical emission^34^ and health effects^35^. In one study, waterpipe tobacco smoke was generated using three commercially available electric heating elements for tobacco smoking and researchers compared CO, PAH, nicotine and aldehyde emissions with waterpipe tobacco smoke generated by charcoal heating^34^. In the other study, the effect of waterpipe tobacco smoke heated by charcoal or electrically on endothelial function was measured in healthy human subjects^35^. Both studies showed that replacing charcoal with an electric heating element produced similar amounts of nicotine, but greatly reduced CO in waterpipe tobacco smoke^34,35^. On the other hand, electrical heating greatly increased the amount of volatile aldehydes including acrolein, a compound associated with respiratory disease^34^. The authors hypothesized that the constant power output of electrical heating devices may cause a greater continuous heating of the tobacco compared to charcoal heating. As a consequence, greater thermal degradation of the tobacco may occur, resulting in increased generation of volatile aldehydes^34^. Furthermore, where CO generated from charcoal acted as a vasodilator molecule masking endothelial disfunction in healthy human waterpipe tobacco smokers, electrical heating did not show this masking effect, causing impaired endothelial function in the same manner as was observed with cigarette smoking^35^. Thus, the reviewed studies show that charcoal as well as electrical heating sources cause significant increases in different but toxic compounds, and equally contribute to the adverse health effects associated with WPS.

### Device components

The systematic review revealed two studies that address the effect of specific device components on toxicity of WPS^37,38^. These studies concern the fact that waterpipes are often smoked in social settings and it is a common practice to share the waterpipe with other smokers. The systematic search revealed no studies reporting on the attractiveness or addictiveness of device components in WPS.

As such, friends may share one mouthpiece during a joint event of waterpipe smoking. Also, employees of the waterpipe establishment may use the same mouthpiece when preparing the waterpipe. This shared practice of waterpipe smoking is associated with the transmission of pathogens, including bacteria and viruses. Transmission of infectious diseases also could be due to poor sanitation and inadequate cleaning of waterpipe devices in public settings. A suggestion proposed by the authors is the use of a personal mouth tip in waterpipe venues. The effectiveness of using a mouth tip in reducing the harm of sharing a waterpipe with others has not been determined^37^.

Another study reports on user toxicant exposure of CO and nicotine when sharing a waterpipe (so-called dyad sessions). The rationale of this study is that measurement of toxicant exposure in dyad sessions is more realistic as waterpipe is often used in a group setting, compared to toxicant exposure of individual waterpipe users (singleton sessions) that are most commonly reported^38^. The study shows that the nett exposure to CO and nicotine is similar and significant in both dyad and singleton sessions. Considering the lower inhalation volume in dyad sessions, this means that compared to singleton sessions, dyad sessions have a greater toxicant intake^38^.

### Packaging, warning, and labelling

The systematic search yielded 12 studies reporting on packaging, warning, and labelling of either waterpipe products or the waterpipe itself. These factors are all related to the attractiveness of WPS.

Studies revealed that packs are considered less attractive and health warnings are more impactful as health warnings increase in size and packaging becomes less branded^39^; larger warnings in plain packaging instead of colorful packages are consider more effective in preventing initiation and stimulating smoking cessation^40^.

Health warning labels are effective in communicating health risks associated with waterpipe smoking^41,42^, motivating waterpipe smokers to think about quitting^42-44^ or reducing their consumption^42^, and motivating non-smokers to remain smokefree^42^. Two studies among college students smoking waterpipe showed that text-only messages and pictorial labels warning about harm to (unborn) children were the most effective in motivating waterpipe smokers to think about quitting^43,44^. Another study among adolescents and young adults (both users, and susceptible non-users) argued that adolescents and young adults underestimate and discount the long-term risks associated with waterpipe use, and therefore effective messages may be those that focus on the acute/immediate health and cosmetic effects^45^. Another study also showed that warnings featuring more immediate effects such as harmful effects on teeth were the most effective^40^. According to an expert panel of waterpipe scientists, the most important waterpipe-specific health warnings include labels on oral and heart disease, harmful effects on new-born children and the amount of smoke inhaled from WP compared to cigarettes^46^.

Unfortunately, warnings are not always common practice, and packages may also contain misleading information. A study in India found that barely 6% of the waterpipe products sold online displayed any age-specific or health-related warning, and close to 20% of the products were described with words such as herbal and safer than cigarettes since they claim to be selling sweetened and flavored non-tobacco products for use in waterpipes^47^. Packages collected at an international fair showed all packaging information on constituents and emissions to be misleading, and that the majority of charcoal products displayed environmentally friendly descriptors and/or claims of reduced harm^48^.

Furthermore, users in a lounge are generally not exposed to waterpipe tobacco-pack health warnings, as the waterpipe is already prepared by the staff^39,41^. Consequently, health warnings at the point-of-consumption had more reported impact than health warnings at the point-of-sale^39^, and customers suggested to place it on the waterpipe device itself, e.g. on the mouthpiece^41^ or the pipe handle^44^, or base, mouthpiece and stem^43^. Placing pictorial health warning labels on the waterpipe device has been shown to be effective in increasing harm perception, reducing a smoker’s positive experiences, puffing parameters, and exposure to harmful respiratory toxicants such as CO^49^. Another option is including a health warning in café waterpipe menus^41^, including online waterpipe menus that are also used to promote the waterpipe products^21^. Indeed, participants presented with waterpipe lounge menus, each with three fruit-flavored options and one tobacco flavored option, exposed to a health warning were more likely to opt out^22^.

## DISCUSSION

Waterpipe smoking using sweetened, flavored products has become a widespread global phenomenon^3^, and the number of waterpipe smokers is rising rapidly^50,51^. While there is a widely held misconception that waterpipe smoking is not harmful to health, it is associated with a variety of adverse health outcomes on both a short- and long-term basis^50,51^. Waterpipe smoke contains significant concentrations of toxicants thought to cause dependence, heart disease, lung disease and cancer in cigarette smokers, and includes 27 known or suspected carcinogens^3^. Also, waterpipe smoking supports nicotine and tobacco dependence^4^. Hence, to decrease harmful health effects associated with waterpipe use, it is important to reduce its attractiveness, addictiveness, and toxicity. Product regulation, as advised by Article 9 of the FCTC, is an important tool in this respect. Here, we discuss our findings regarding waterpipe products, heating sources, device components, and packages, and make recommendations for regulation of these waterpipe product characteristics for the protection of public health.

### Reflection on main findings

Waterpipe product regulation is challenging as a waterpipe set-up is not one single product such as a cigarette, but consists of three distinct parts: waterpipe tobacco, the heating source, and the device. The device itself also contains multiple components, such as the hose, mouthpiece, base, and liquid^12^. Many different parts and components can be combined to create unique set-ups with different effects on consumer appeal and exposure. Regarding specific waterpipe product characteristics that influence human health, most of the data we found affect product attractiveness via flavors. Only few data covered the differential effects of specific waterpipe characteristics on addictiveness or toxicity.

#### Characteristics of waterpipe products to influence human health

Our systematic review showed that flavor is the main characteristic to attract smokers to start and continue waterpipe smoking, already at a young age^18-20^. Increased liking of tobacco flavor stimulates use, resulting in nicotine dependence. This prevents smoking cessation and may induce a gateway effect towards cigarette smoking. Other characteristics of WPS that increase attractiveness are the price of the tobacco product and nicotine content^21,22^. In addition, the socializing aspect of waterpipe smoking may positively influence attractiveness. This includes the group activity of waterpipe smoking as well as modern online social communities that attract consumers to waterpipe smoking events^18,23,26^.

Our findings are corroborated by others. The waterpipe molasses create unique regulatory challenges, ranging from the great variety of flavors appealing to youth to the use of herbal products, claimed to be tobacco- or nicotine-free^12^. A systematic review toward flavored products suggests that flavored tobacco use is associated with young age and that consumers may perceive flavored products more favorably than non-flavored products^52^. Another systematic review on flavored products, including waterpipe, concluded that the majority of studies reported positive perceptions of flavored products, particularly among young adults and adolescents, with beliefs that flavored products are less harmful than cigarettes, and that flavors specifically led to experimentation and/or initiation of flavored products^53^.

Most adolescents experimenting with tobacco started with a flavored product, and most current adolescent tobacco users reported use of flavored products^54^. Among adolescent past 30-day waterpipe smokers, the majority reported liking the flavors available as a reason for use^12^. In samples of four Eastern Mediterranean countries, participants preferred fruit-flavored varieties to tobacco flavor, and lower nicotine content. Flavor accounted for over 80% of waterpipe smoking decisions^55^.

These findings provide support for regulating flavors in most tobacco products. In the EU TPD^11^, cigarettes and roll-your-own (RYO) tobacco are not allowed to have characterizing flavors other than one of tobacco, and the Family Smoking Prevention and Tobacco Control Act banned cigarettes containing characterizing flavors other than tobacco or menthol^50,51^. Tobacco smoked in water pipes regularly comes in characterizing flavors such as fruit, mint, and vanilla^50^. Given that these products are also particularly popular among young people, regulators should consider to implement a characterizing flavors ban for waterpipe products.

Studies on toxicity of unburned products, waterpipe smoke, indoor air of waterpipe cafés and water waste from waterpipes show that consumers, bystanders and potentially the environment are exposed to nicotine, tar, CO, trace metals, PAHs, aldehydes, and ultrafine particles^27-32,56^. Levels of exposure to these constituents by waterpipe smoking is in many cases comparable to exposure in cigarette smoking, and, besides nicotine, no differences between herbal and tobacco products for waterpipe exist. Furthermore, exposure is at levels that are high enough to increase risks for cancer and non-cancer hazards.

The reviewed studies all relate to the known risks associated with the constituents either found in unburned tobacco, waterpipe tobacco smoke, indoor air, or environmental water waste, based on e.g. internationally agreed exposure limits and classification by the International Agency for Research on Cancer (IARC). However, there is no detailed risk assessment on specific toxicants and toxicant classes that allow for discrimination of effects of tobacco products or product constituents on tobacco-related health hazards.

#### Heating sources

Heating sources for waterpipe tobacco include charcoal and electrical heating. This systematic review shows that advantages and disadvantages of both heating sources exist, in both attractiveness and toxicity. Visitors of waterpipe smoking establishments prefer the use of charcoal and even more specifically, high quality charcoal. Visitors dislike quick-light charcoal because of its chemical flavour^21^. Charcoal is a known source of CO and various toxicants associated with a range of human health effects^33^. Electrical heating may significantly decrease CO production, but, due to the constant heating, higher levels of aldehydes are produced, which are associated with adverse health effects as well^34,35^. In conclusion, from a regulatory perspective, no ‘safe’ heating source for WPS can be advised.

#### Device components

Waterpipes are available in many designs, types and shapes. Theoretically, components that enhance the waterpipe appearance, thereby increasing its attractivity and popularity, may stimulate use and hence exposure to toxic constituents in WPS. In this systematic review, only two studies were retrieved that discussed the influence of device components on human health. Both studies were concerned with the social activity of group WPS. The first study reported on the transfer of infectious diseases as an adverse health effect that may occur after sharing a single mouthpiece in group WPS. The second study investigated toxicant exposure after single or dual waterpipe smoking^37,38^. In conclusion, group waterpipe smoking not only increases the attractiveness in the social experience of waterpipe smoking but also influences the toxicant and other health hazard exposures (bacteria, viruses) that may result in adverse health effects.

#### Packaging, warning, and labelling

Changing the package itself, by making it more generic and less attractive, and adding a health warning, is an option for policy makers to discourage waterpipe use. A systematic review on international practices in controlling WPS showed that almost all of the waterpipe products and accessories do not comply with the regulations on health warning labelling practices as stipulated under Article 11 of WHO FCTC^57^. Waterpipe tobacco packages, retailer and waterpipe café websites often use words (‘natural’), claims (‘0% tar’), and images (fruits, desserts) that may affect harm perception and product appeal^12^. Exposure to warnings increases perceptions of waterpipe risk^58^. Three countries (Israel, Lebanon, and Turkey) have set specific laws on health warnings regarding WPS^50^. The warnings can be adapted based on the context and policy landscape of the target country and can be further fine-tuned based on feedback from WP smokers and non-smokers^46^. Because much of WPS occurs in commercial waterpipe cafés and bars, where employees prepare the waterpipe for customers, or at social gatherings, users may rarely be exposed to the packaging^12^. Thus, adaptation of labelling and health warning approaches for waterpipes is needed to effectively communicate potential health risks to consumers, such as on the waterpipe tobacco menu in a café setting, and various parts of the waterpipe device (e.g. water bowl, stem)^59^. For this reason, in Turkey, it is mandatory to place warning labels on bottles and bowls of waterpipes at public places^10,50,60^. Obviously, this would not be necessary if waterpipe smoking at public places is included in smoking bans.

### Potential policy implications

Overall, regulatory frameworks for waterpipe establishments should address the complex context of WPS including the venue (i.e. physical, website, menu), the smoking products, the device/accessories, and charcoal^21^. An overall framework should identify regulatory targets for waterpipe product components (i.e. tobacco/herbal products, charcoal, and device), the waterpipe café setting, and its marketing environment dominated by Internet promotion^59^. For instance, in the USA, Section 101 of the FSPTCA defines a ‘tobacco product’ to include ‘any component, part, or accessory’ of that product^12^.

Based on the evidence we found, policy makers are advised to consider the following recommendations for regulation:

1. Include non-tobacco products with or without nicotine in tobacco product regulations and smoking bans.
  As waterpipe molasses may not always contain tobacco and/or nicotine, it may not be regulated by existing tobacco product regulation laws. Still, such products have harmful health effects similar to those of tobacco-containing molasses for users and bystanders. Examples include herbal molasses or steam stones.
2. Ban waterpipe molasses with strong characterizing flavors or ban the use of flavorings at any level.
  Waterpipe molasses usually contain large amounts of flavorings resulting in strong characterizing flavors other than one of tobacco. As such flavors are particularly attractive to youth, bans on such products should be considered. The EU and the USA already ban cigarettes with characterizing flavors. In Brazil and Canada, use of flavorings per se is forbidden, also flavorings at lower levels.
3. Require dissemination of information on all waterpipe tobacco elements to the national regulator.
  Manufacturers should disclose the ingredients and contaminants of tobacco and charcoal products marketed for waterpipe use (including tobacco or herbal molasses, waterpipe stones and other products intended for mixing with tobacco or charcoal), as well as waterpipe device components (e.g. hose infiltration) and accessories (e.g. mouthpieces).
4. Communicate that there is no safe option for waterpipe use
  Risk communication should address misperceptions, for instance that flavored products are less harmful to health. Using any type of waterpipe is harmful to health, even though some products may be even more harmful than others.
5. Prescribe testing to regulate contents of waterpipe smoking products.
  Regarding contents, consider testing to regulate nicotine, humectants (existing WHO Tobacco Laboratory Network methods for cigarette filler can be adapted to this purpose), sugars and flavors. Regulating these contents can reduce attractiveness and addictiveness of waterpipe use. However, regulators need to realize that although reducing attractiveness and addictiveness may prevent use and stimulate cessation at the population level, it does not eliminate the harmful effects of WPS at the product level. This also depends on the effects on the entire matrix of toxic emissions.
6. Stimulate research on emissions of waterpipes.
  Emissions depend not only on the waterpipe product smoked but also on the combination of waterpipe product, charcoal type, waterpipe design, waterpipe preparation method, puff topography and their interactions. For the regulation of waterpipe emissions, waterpipe smoking topography is an important aspect of waterpipe use patterns and may differ at the local, country and regional level. However, regulators need to realize that even if clear evidence is found that specific product characteristics can be modified to decrease the levels of certain toxic emissions in smoke, this does not necessarily mean that the harmful health effects of the product will be lower. This also depends on the effects of the entire matrix of toxic emissions and the effects of the product characteristic on product use.
7. Prescribe a warning on waterpipe device, on the menu or on the entrance of the lounge.
  As users often do not see the package, since waterpipes are prepared by lounge personnel, it is recommended to oblige a warning on the waterpipe device and/or the menu, and/or the lounge entrance. Obviously, it would not be necessary to include warnings on the menu or in lounges if waterpipe smoking at public places is already included in smoking bans (see Recommendation 1). Warnings on devices and strengthened tobacco control measures can play a role in limiting the spread of infectious diseases such as COVID-19.

### Strengths and limitations

To the best of our knowledge, our review is the first to provide an overview on the influence of specific waterpipe product characteristics on human health, in particular WPS attractiveness, addictiveness, and toxicity for users and bystanders. In order to protect public health, we translate our findings to evidence-based, specific recommendations for regulating waterpipe products, heating sources, device components, and packages. Regarding limitations, we did not follow a weight of evidence approach, but simply reported on the findings of all studies from our search. Also, it needs to be realized that most studies have been performed in the Middle East or in the United States, implying that there may be a cultural bias, especially in the studies that address attractiveness of WPS. Consequently, findings may not be directly translatable to other regions or countries such as Europe. Finally, the lack of available evidence, on the effect of specific waterpipe characteristics on toxicity and addictiveness, means that we could not give any specific recommendations on these topics.

## CONCLUSIONS

Our review on the effect of waterpipe product characteristics shows that variations in waterpipe products, including heating sources, device components, and packages, influence attractiveness, addictiveness, and toxicity for users and bystanders. Based on our findings, we made a number of recommendations for regulation of these waterpipe product characteristics for the protection of public health, that are of relevance to policy makers in the context of the legislations in their countries or regions. The recommendations from this systematic review are in line with the FCTC and the WHO advisory note on waterpipe tobacco smoking^61^. We advise to include non-tobacco products with or without nicotine in tobacco product regulations and smoking bans, ban waterpipe molasses with strong characterizing flavors, or ban the use of flavorings at any level, require dissemination of information on all waterpipe tobacco elements to the national regulator, prescribe testing to regulate contents of waterpipe products and heating sources, stimulate research on emissions of waterpipes, and prescribe warnings placed on the waterpipe device, on the menu, or on the entrance of the WPS lounge.
