# Supplementary material for: Options for waterpipe product regulation: A systematic review on product characteristics that affect attractiveness, addictiveness and toxicity of waterpipe use
Source: Tob Induc Dis. 2020 Aug 25;18:69. doi: 10.18332/tid/125079 (PMC7485441; doi:10.18332/tid/125079)
Supplement: Supplementary file 1 [file TID-18-69-S1.pdf]

Supplementary file, Table S1

#41	#35 OR #39	423
#40	#34 OR #38	265
#39	#36 AND 'review'/it AND [1950-2015]/py	23
#38	#36 AND 'review'/it AND [2016-2019]/py	10
#37	#36 AND 'Review'/it	33
#36	#13 NOT #32	802
#35	#13 AND #32 AND [1950-2015]/py	400
#34	#13 AND #32 AND [2016-2019]/py	255
#33	#13 AND #32	655
#32	#14 OR #15 OR #16 OR #17 OR #21 OR #22 OR #23 OR #24 OR #25 OR #26 OR #27 OR #28 OR #29 OR #30 OR #31	5,939,467
#31	'adverse event'/exp	596,461
#30	'warning*':ti	7,900
#29	'packaging'/exp OR 'packag*':ti	34,348
#28	'water vapor'/exp OR stone:ti OR stones:ti	34,696
#27	'coal'/exp OR 'coal tar'/exp OR 'coal*':ti	21,358
#26	'herbal medicine'/exp OR 'herba*':ti	32,654
#25	'molasses'/exp OR 'molasses*':ti	2,103
#24	'practice guideline'/exp	490,032
#23	'policy'/exp OR 'policy':ti	287,117
#22	'regulatory mechanism'/exp OR 'regulation*':ti OR 'regulator*':ti	1,218,345
#21	'flavor'/exp OR 'flavor*':ti OR 'flavour*':ti	8,595
#20	#13 AND #18 AND [2016-2019]/py	212
#19	#13 AND #18	554
#18	#14 OR #15 OR #16 OR #17	3,775,002
#17	'risk*':ti OR 'danger*':ti OR 'toxic*':ti	795,367
#16	'danger, risk, safety and related phenomena'/exp	2,814,085
#15	'toxicity and intoxication'/exp	912,384
#14	'risk'/exp	2,256,980
#13	#1 OR #2 OR #3 OR #4 OR #5 OR #6 OR #7 OR #8 OR #9 OR #12	1,457
#12	#10 AND #11	170
#11	waterpipe*:ti,ab OR 'water pipe*':ti,ab OR hookah*:ti,ab OR shisha*:ti,ab	2,378
#10	'smokeless tobacco'/exp	4,585
#9	shisha*:ti	143
#8	'hookah*':ti	323
#7	'hookah'/exp	242
#6	'water pipe*':ti	319
#5	waterpipe*:ti	493
#4	'waterpipe'/exp	19
#3	'waterpipe tobacco smoking'/exp	51
#2	'waterpipe smoking'/exp	95
#1	'waterpipe tobacco'/exp	103

© 2020 Kienhuis A.S. and Talhout R.
